# Reduced Intestinal Tumorigenesis in APCmin Mice Lacking Melanin-Concentrating Hormone

**DOI:** 10.1371/journal.pone.0041914

**Published:** 2012-07-27

**Authors:** Jutta M. Nagel, Brenda M. Geiger, Apostolos K. A. Karagiannis, Beatriz Gras-Miralles, David Horst, Robert M. Najarian, Dimitrios C. Ziogas, XinHua Chen, Efi Kokkotou

**Affiliations:** Division of Gastroenterology, Beth Israel Deaconess Medical Center, Harvard Medical School, Boston, Massachusetts, United States of America; University Claude Bernard Lyon 1, France

## Abstract

**Background:**

Melanin-concentrating hormone (MCH) is an evolutionary conserved hypothalamic neuropeptide that in mammals primarily regulates appetite and energy balance. We have recently identified a novel role for MCH in intestinal inflammation by demonstrating attenuated experimental colitis in MCH deficient mice or wild type mice treated with an anti-MCH antibody. Therefore, targeting MCH has been proposed for the treatment of inflammatory bowel disease. Given the link between chronic intestinal inflammation and colorectal cancer, in the present study we sought to investigate whether blocking MCH might have effects on intestinal tumorigenesis that are independent of inflammation.

**Methodology:**

Tumor development was evaluated in MCH-deficient mice crossed to the APCmin mice which develop spontaneously intestinal adenomas. A different cohort of MCH−/− and MCH+/+ mice in the APCmin background was treated with dextran sodium sulphate (DSS) to induce inflammation-dependent colorectal tumors. In Caco2 human colorectal adenocarcinoma cells, the role of MCH on cell survival, proliferation and apoptosis was investigated.

**Results:**

APCmin mice lacking MCH developed fewer, smaller and less dysplastic tumors in the intestine and colon which at the molecular level are characterized by attenuated activation of the wnt/beta-catenin signaling pathway and increased apoptotic indices. Form a mechanistic point of view, MCH increased the survival of colonic adenocarcinoma Caco2 cells via inhibiting apoptosis, consistent with the mouse studies.

**Conclusion:**

In addition to modulating inflammation, MCH was found to promote intestinal tumorigenesis at least in part by inhibiting epithelial cell apoptosis. Thereby, blocking MCH as a therapeutic approach is expected to decrease the risk for colorectal cancer.

## Introduction

Melanin-concentrating hormone (MCH) is an evolutionarily conserved 17- to 19-amino-acid cyclic neuropeptide which was first recognized for its role in the aggregation of skin melanosomes in teleost fish in response to an environmental threat. In the last decade, important functions for MCH in mammals have emerged including the regulation of food intake, sleep, anxiety and glucose metabolism [1]. However, as of today, the focus on MCH research has been on its centrally mediated effects and, among one thousand publications concerning MCH, only twenty address aspects of its peripheral actions, an area which remains largely unexplored. This is despite reports documenting the presence of MCH in peripheral tissues and organs, including the intestine and colon [2], [3], [4]. Our own data also demonstrate MCH immunoreactivity in rat myenteric and submucosal plexus [5].

We have recently reported that mRNA expression of MCH and MCHR1 are upregulated in the afflicted mucosa of patients with inflammatory bowel disease (IBD) [5]. Most importantly, mice lacking MCH were found to be protected from acute experimental colitis [5] and *C. difficile* toxin-A mediated ileitis [6], suggesting a proinflammatory role of MCH. At the molecular level, we discovered that in colonic epithelial cells, MCHR1 expression is upregulated in response to an inflammatory milieu [5], [6]. In turn, in the same cells activation of MCHR1 stimulated the expression of various proinflammatory cytokines and chemokines [5], [6], most likely by activating erk1/2 and NFkB, as has been described for the neuropeptides substance P and neurotensin [7], [8].

In the present study, we investigated whether MCH might play a role in intestinal tumorigenesis based on a) the expression of MCHR1 on colonocytes, b) our previous studies indicating the proinflammatory properties of MCH in the intestine, [5], [6], and c) the well-established link between inflammation and cancer [9]. We first examined expression of MCHR1 on human colonic adenocarcinomas and in mouse LGR5 positive intestinal stem cells. Subsequently, we tested the effects of MCH on the survival and proliferation of Caco2 colonic epithelial cells. The impact of MCH ablation in intestinal tumor development was interrogated using APCmin mice, a model which shares genetic and phenotypic similarities to human colorectal neoplasia [10], [11]. Notably in humans, mutations in the APC gene represent an initiating factor in the process of colon carcinogenesis and can be detected in the vast majority of familial adenomatous polyposis (FAP) patients, and in 60–80% of sporadic colorectal tumors [12].

In APCmin mice, a mutation in the *apc* gene prevents the APC complex from binding to and phosphorylating beta-catenin, a downstream effector of the wnt signalling pathway [10], [13]. As a consequence, beta-catenin, instead of being marked for proteosomal degradation, is transported to the nucleus where it activates transcription of target genes, including the oncogene c-myc [14], [15]. Depending on the genetic background and additional environmental factors, APCmin mice develop 30–50 intestinal adenomas, predominantly in the distal part of the small intestine [16], [17], [18]. However, in response to a colonic irritant like DSS, which triggers an inflammatory response, these mice develop large colonic tumors, in particular close to the rectum, well in advance of the development of tumors in their small intestine [19]. In our study we used both of these models and we obtained consistent results suggesting an inflammation-independent tumor promoting effect of MCH.

## Materials and Methods

### Ethics Statement

All studies were carried out according to the recommendations in the Guide for the Care and Use of Laboratory Animals of the National Institutes of Health and approved by the Beth Israel Deaconess Medical Center’s Institutional Animal Care and Use Committee. To minimize suffering, mice were euthanized prior to tissue harvesting.

### Mice

MCH+/− mice were crossed with APCmin/+ mice (Jackson Laboratories, Bar Harbour, Maine), both of the C57BL6 background, to yield the following compound genotypes: MCH+/+APCmin/+ and MCH−/−APCmin/+, (described herein for simplicity as WT and MCH-KO, respectively). Genotyping for MCH was performed as previously described^10^. Genotyping of APCmin mice was performed using the standard PCR protocol provided by Jackson Laboratory. LGR5 (leucine-rich-repeat-containing G-protein-coupled receptor 5)-labeled mice (Lgr5tm1(cre/ERT2)Cle/J) were purchased from Jackson Laboratories. Mice were maintained in a controlled environment with a 12 h light/dark cycle, and unlimited access to food (standard rodent chow) and water.

### Intestinal Tumor Development in APCmin Mice Lacking MCH

Male MCH+/+APCmin/+ (WT) (n = 15) and MCH−/−APCmin/+ (MCH-KO) mice (n = 18) were sacrificed at 14 weeks of age and the small intestine was divided in three equal segments: proximal, medial and distal. After cleaning the small intestines by briefly washing with ice-cold PBS, tumors in each segment were counted and measured macroscopically by the same investigator using an electronic calliper (VWR). If necessary, lesions were verified using a dissecting microscope (Bausch & Lomb, Stereozoom 5, zoom range 0.8–4.0). Tissues were fixed overnight in 10% buffered formalin and the distal third of the small intestine was prepared as Swiss rolls. Paraffin-embedded H&E stained tissue sections were used for histological analysis.

### DSS-induced Colorectal Tumor Development in APCmin Mice Lacking MCH

Colitis was induced in 6-week old female MCH+/+APCmin/+ (WT) (n = 24) and MCH−/−APCmin/+ (MCH-KO) mice (n = 32) by 7 days of treatment with 2% (w/v) Dextran Sulfate Sodium (DSS, MP Biomedicals, Solon, OH, USA) dissolved in their drinking water ^9^. Mice were evaluated for colonic tumor development at 11 weeks of age. Colons were briefly rinsed with ice-cold PBS. Lesions were counted and measured macroscopically as described above. Tissues were then fixed overnight in 10% buffered formalin and prepared as modified Swiss rolls. Paraffin-embedded specimens were cut to 5-micrometer sections and stained with hematoxylin and eosin (H&E) for histological analyses.

### Immunostaining

Immunostaining was performed in paraffin sections by the Specialized Histopathology Core at the Dana-Farber/Harvard Cancer Center. Antigen retrieval was achieved by EDTA in all cases except p-erk1/2 where citrate was used. Following endogenous peroxidase and protein blocking (Dako), the primary antibodies for Ki67 (Vector Labs cat#VP-RM04 Rabbit polyclonal, 1∶250 dilution), beta-catenin (BD Pharmingen cat#610154 Mouse Monoclonal, 1∶150 dilution), c-myc (Epitomics cat#1472-1 Rabbit monoclonal, 1∶1000 dilution with TSA kit from PerkinElmer) and p-erk1/2 (Cell Signaling Technologies cat#4370, 1∶150 dilution) were added for 1 hour followed by incubation with secondary labeled anti-mouse or anti-rabbit antibodies and color visualization (En Vision kit, DAKO). Apoptotic cells were labeled with TdT (1∶16 dilution) using the ApopTag peroxidase *in situ* apoptosis detection kit (Millipore cat#S7100). Images were taken using a Zeiss Imager 1 at 10× magnification and scoring of the staining was performed by an investigator blinded to the group assignments.

In studies involving analysis of MCHR1 expression, we used a rabbit polyclonal anti-MCHR1 antibody. This antibody was raised against a conserved epitope in human, rat and mouse sequences and in an *in vitro* functional assay was found to block MCH-mediated inhibition of intracellular cAMP levels [6]. In immunohistochemical studies, the MCHR1 positive neurons identified using this antibody in brain and the enteric nervous system had the expected distribution [5]. Most importantly, treatment of mice with this anti-MCHR1 antibody resulted in attenuation of intestinal inflammation [6]. MCHR1 expression was examined in formalin-fixed intestinal frozen sections of patients with colorectal adenocarcinoma and controls (n = 4 per group, obtained from Origene), of APCmin mice with intestinal tumors, and of Lgr5tm1(cre/ERT2)Cle/J mice. MCHR1 expression was also evaluated in human (Caco2) and murine (MCA-38) colon adenocarcinoma cells grown in coverslips. Slides were incubated with anti-MCHR1 or pre-immune IgG serving as a negative control (1∶200 in Protein Block-Dako) for 30 min at room temperature, followed by incubation with an Alexa Fluor-488 (green) or Alexa Fluor-546 (red) anti-rabbit secondary antibody (Invitrogen), 1∶2000 in Protein Block, for 30 min at room temperature. Slides were mounted using Prolong Gold antifade with DAPI (Invitrogen). Images were taken using a Zeiss LSM510 META confocal system at 40× magnification.

### Cell Treatments

Caco2 human colorectal adenocarcinoma cells were obtained from American Type Culture Collection. Cells growing in complete media (20% FBS) at 60–70% confluence were serum starved (2% FBS) overnight and then incubated for 48 hrs in media containing 2% FBS in the presence of various treatments: MCH (2.4 µg/ml, Bachem Bioscience), IGF-1(10 nM), MCH plus IGF-1, or 20% FBS as a positive control. Cell viability was measured using the MTT cell proliferation assay (ATCC). Cell proliferation was measured by BrdU incorporation using a commercially available kit (Cell Proliferation Elisa, BrdU; Roche Diagnostics). Cell apoptosis was evaluated using the Apo-One Homogenous Caspase3−/7 kit (Promega). Each experiment was performed in six replicas and was repeated twice. MCA-38 murine colon cancer cells were obtained from Dr Nicholas P. Restifo (National Cancer Institute) and cultured in RPMI 1640 media supplemented with 10% fetal calf serum and 1% antibiotic/antimycotic (Invitrogen).

### Western Blot Analysis

NCM460 non-transformed human colonic epithelial cells stably expressing MCHR1 (NCM460/MCHR1) were maintained in M3D media (INCELL) supplemented with 10% fetal bovine serum and 1% antibiotic/antimycotic (Invitrogen). At 80% confluence, cells were serum starved for 16 hrs and then treated with MCH (2.4 µg/ml) for the indicated time points. Cells were harvested in 3× SDS sample buffer (Cell Signalling Technology) and protein concentration in the cell lysates was determined using the DC protein assay (Biorad). Thirty microgram of cell extracts were separated by SDS-PAGE and transferred to a PVDF membrane (Millipore). After blocking, membranes were incubated with anti-phospho erk1/2 ((Thr202/Tyr204)(Cell Signaling Technology) antibody or anti-GAPDH antibody (Santa Cruz Biotechnology) overnight at 4°C, followed by incubation with a peroxidase labeled secondary antibody (Santa Cruz Biotechnology). Specific bands were visualized in film after incubation with the SuperSignal West Pico chemiluminescent substrate (Pierce).

### Statistical Analysis

Results are expressed as group mean +/− SΕM. Data were analyzed by StatView, using unpaired t-test, ANOVA factorial followed by Fisher’s PLSD analysis for multiple comparisons or a chi-square test where appropriate. A level of p<0.05 was considered statistically significant.

## Results

### MCHR1 is Expressed by Intestinal Epithelial Cells, Colonic Adenocarcinoma and Intestinal Crypt Stem Cells

**Figure 1:**
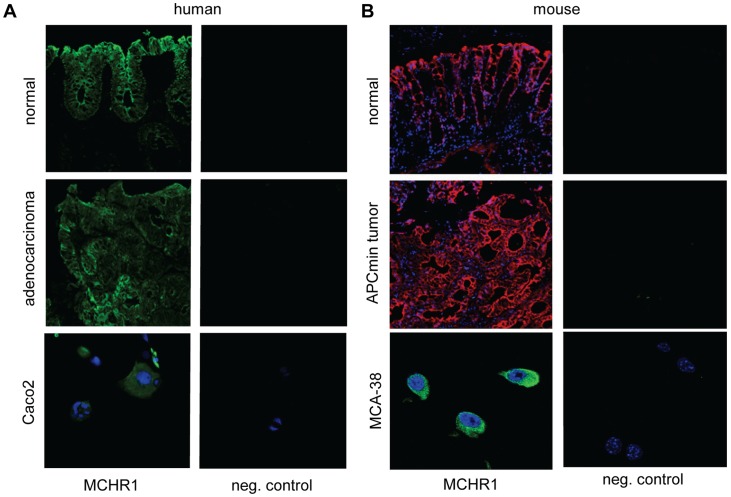
MCHR1 expression in normal colonic epithelial cells and colon adenocarcinoma. (A) In human colonic biopsies, normal epithelium as well as adenocarcinoma cells were positive for MCHR1 staining. Moreover, Caco2 cells, a human colon adenocarcinoma cell line, showed MCHR1 immunoreactivity. (B) Likewise, MCHR1 was found to be expressed by mouse intestinal epithelial cells, intestinal tumors developed in APCmin mice and the MCA-38 murine colon cancer cell line. Incubation with pre-immune serum was used as a negative control.

In humans, we detected expression of MCHR1 in normal colonic epithelial cells as well as in colonic adenocarcinoma cells (fig 1A). Furthermore, according to the Human Protein Atlas project, among the 11 colorectal cancer samples examined, 7 showed strong MCHR1 staining of tumor cells and the remaining a moderate staining (http://www.proteinatlas.org/ENSG00000128285/cancer/colorectalcancer). Likewise, MCHR1 was expressed in the normal mouse intestinal epithelium as well as in tumors developed in the APCmin mice (fig 1B). MCHR1 expression was also detected in a human (Caco2) and mouse (MCA-38) colon cancer cell line by immunofluorescence (fig 1, lower panels) as well as by RT-PCR (data not shown).

**Figure 2:**
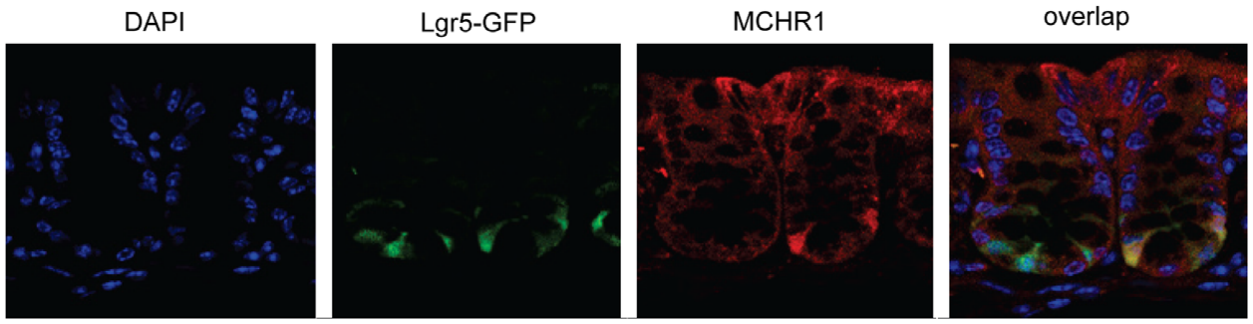
MCHR1 is expressed in LGR5 positive intestinal stem cells. For this experiment we used Lgr5-EGFP-IRES-creERT2 knock-in mice in which expression of EGFP was under the endogenous lgr5 promoter. Colonic biopsies from the above mice were stained with an anti-MCH antibody and co-localization (yellow) of MCHR1 (red) and LGR5 (green) was detected under a fluorescent microscope.

It has been suggested that intestinal adenomas arise from undifferentiated crypt base stem cells [20]. We thus investigated whether these stem cells are also MCHR1 positive. Intestinal crypt stem cells were labeled using a transgenic mouse that expresses eGFP under the LGR5 promoter as previously described [20]. Using an antibody against MCHR1, we demonstrated colocalization of MCH-R1 (red) with GFP-labeled LGR5- positive (green) intestinal stem cells (fig 2).

### MCH Promotes Survival of Colon Adenocarcinoma Cells by Inhibiting Apoptosis

**Figure 3:**
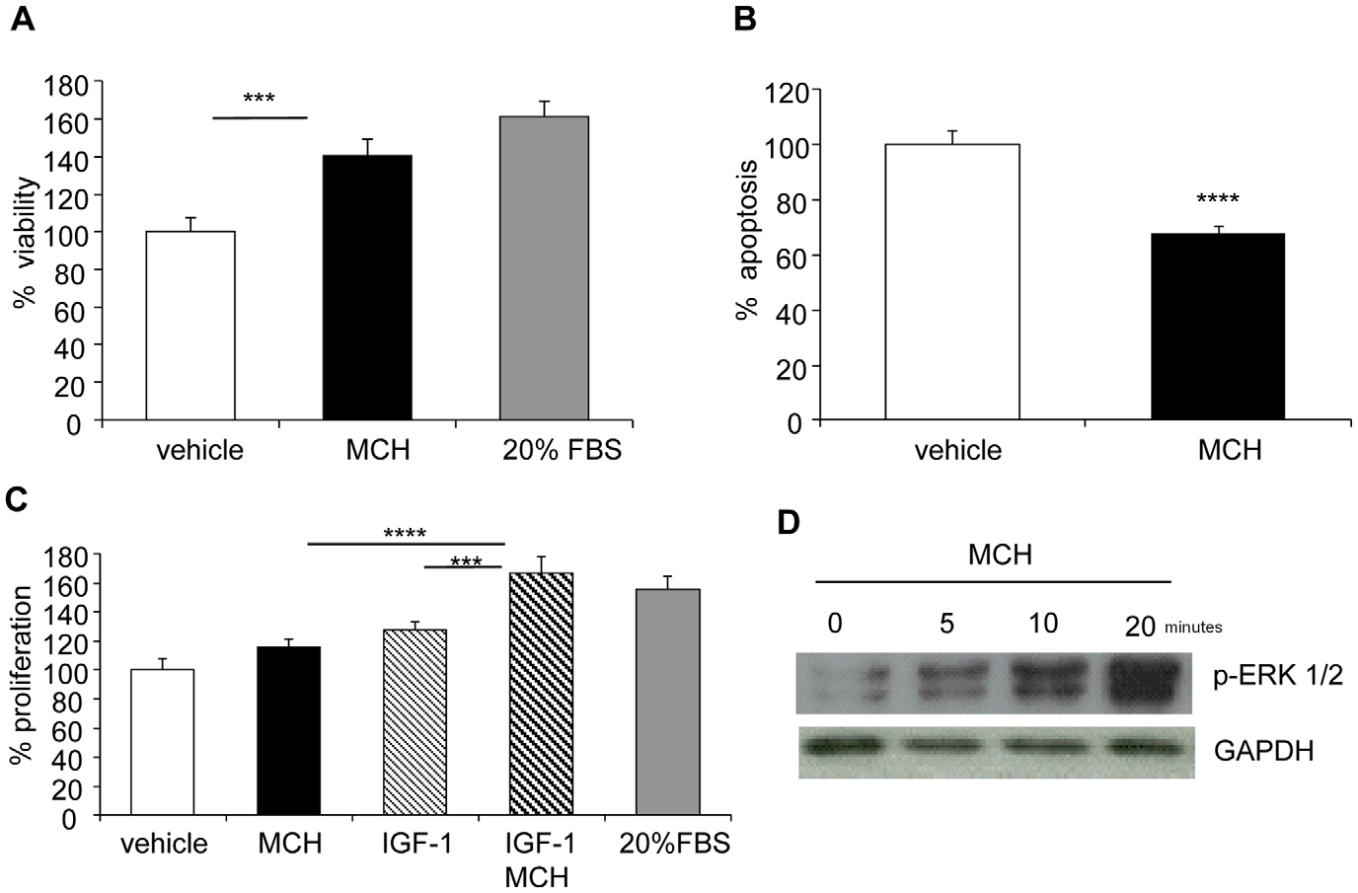
MCH increases cell viability *in vitro* by suppressing apoptosis. Caco2 human colonic adenocarcinoma cells were serum starved overnight (2% FBS) and treated for 48 hrs with MCH (2.4 µg/ml). (A) Cell viability was measured using the MTT assay. Treatment with 20% FBS served as a positive control. (B) Cell apoptosis was measured by the Apo-One Homogenous Caspase3−/7 assay. (C) Cells were treated with MCH (2.4 µg/ml), IGF-1 (10 nM) or their combination and cell proliferation was assessed by measuring BrdU incorporation using a colorimetric assay. Results are expressed relatively to vehicle treated cells (100). Graphs depict mean±sem of 6 replicates and are representative of 2 independent experiments. ***p<0.005; ****p<0.001 (D) NCM460 non-transformed human colonic epithelial cells stably expressing human MCHR1 (NCM460/MCHR1) were treated with MCH and erk1/2 phosphorylation at different time points was analyzed by western blot. Expression of GAPDH serves as a control for equal loading.

Because we had detected MCHR1 in human carcinomas, we next measured the *in vitro* effects of MCH on Caco2 cells. Treatment of these cells with MCH for 48 hours substantially increased cell viability as determined by the MTT dye conversion assay (100±7.2 vs 140.6±8.4, vehicle vs MCH treatment, respectively, p = 0.0029; fig 3A). Two potential mechanisms may account for this observation, namely increased cell proliferation or inhibition of cell death. Indeed, MCH had a strong effect on inhibiting apoptosis in these cells (100±4.9 vs 67.6±2.5, vehicle versus MCH treatment, respectively; p = 0.0004; fig 3B). Under the experimental conditions tested, MCH was not found to independently increase cell proliferation. However, MCH in combination with IGF-1 significantly enhanced cell proliferation (127.6+5.9 vs 166.4+11.8; IGF-1 vs IGF-1/MCH, respectively, p = 0.0026; fig 3C). We can therefore speculate that IGF-1 and other growth factors released in the *in vivo* situation may synergize with MCH to add to their pro-proliferative effect. Treatment with MCH also increased cell survival by inhibiting apoptosis in HT-29 human colon adenocarcinoma cells and in MCA-38 murine colon carcinoma cells (data not shown).

It has been previously demonstrated that signaling via MCHR1 activates erk1/2 in HEK293 cells stably expressing MCHR1 and in 3T3L1 preadipocytes [21], [22]. To test whether MCH had similar effects in colonic epithelial cells, we used a non-transformed human colonic cell line (NCM460). In these cells endogenous MCHR1 expression at baseline is very low, but it can be significantly upregulated in response to inflammation [6]. Thus these cells were stably transfected with human MCHR1 (NCM460/MCHR1), which resulted in a 5–10 fold increase in MCHR1 expression levels. Indeed, treatment of NCM460/MCHR1 cells with MCH induced erk1/2 phosphorylation in a time-dependent manner (fig. 3D).

### MCH-deficiency Protects APCmin Mice Against Intestinal Adenoma Development and Progression

**Figure 4:**
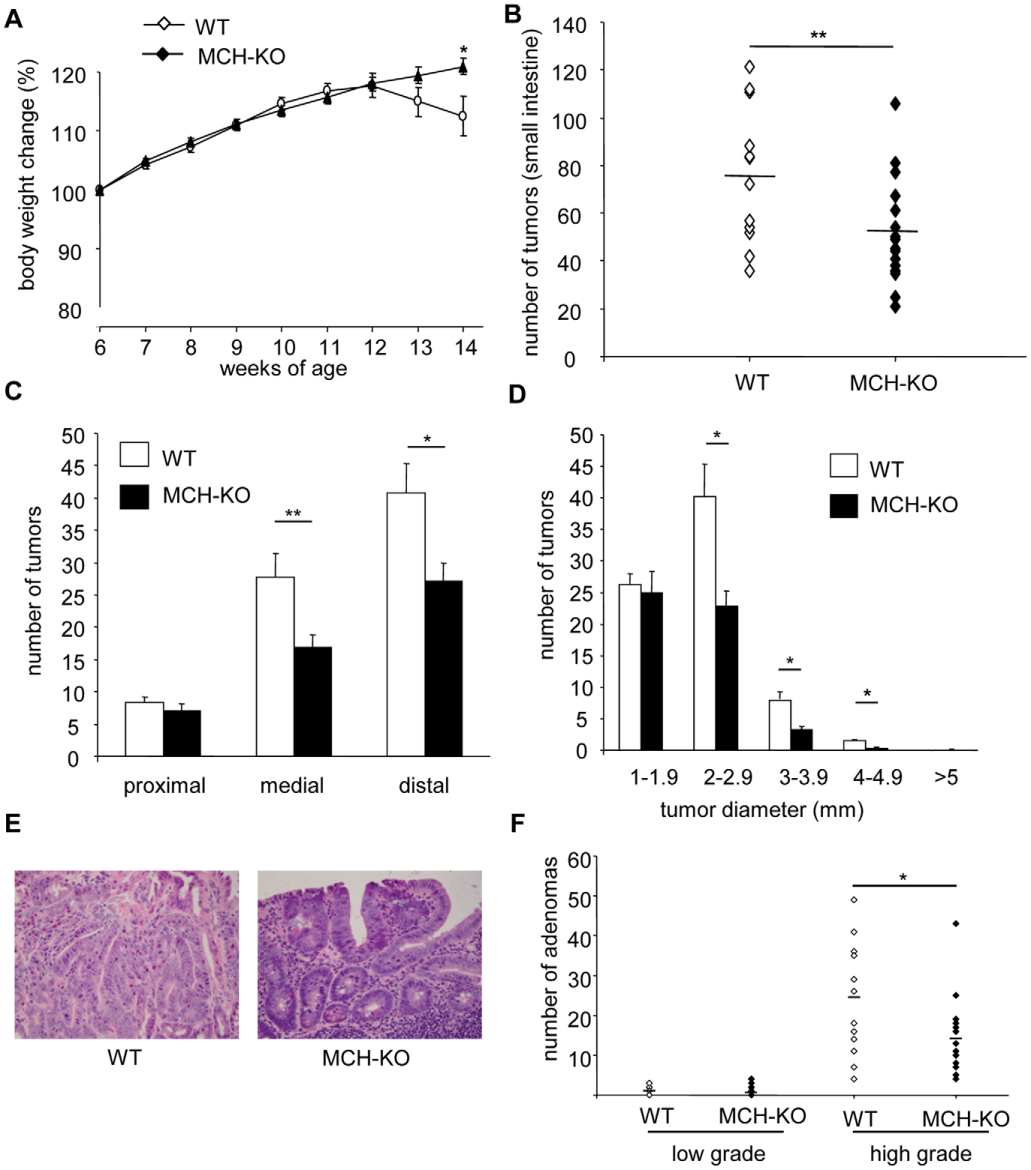
Reduced incidence and size of intestinal adenomas in APCmin mice lacking MCH. Mice of a compound genotype were generated, and were wild type (WT; n = 15) or deficient for MCH (MCH-KO; n = 18), all in the APCmin genetic background. At 14 weeks of age, mice were sacrificed and intestinal tumors were evaluated macroscopically and by histology. (A) Changes in body weight were expressed as % of body weight at 6 weeks of age. (B) Incidence of intestinal tumors in WT and MCH-KO mice. Horizontal bars represent the mean number of tumors per genotype. (C) Incidence of adenomas according to their location in the small intestine. (D) Distribution of intestinal tumors in WT and MCH-KO mice according to their diameter. (E) Representative H&E stained sections of a high grade adenoma in a WT mouse, and a low grade adenoma in a MCH-KO mouse. (F) Incidence of low and high grade adenomas in WT and MCH-KO mice as evaluated in H&E stained sections by a pathologist (RMN). *p<0.05; **p<0.01.

To determine the *in vivo* role of MCH in colorectal cancer, we evaluated spontaneous tumor development in WT (n = 15) and MCH-KO (n = 18) mice of the APCmin genetic background. As shown in fig 4A, WT mice started losing weight around 12 weeks of age, whereas MCH-KO mice continued to gain weight (p = 0.0355 at sacrifice; fig 4A). At 14 weeks of age, MCH-KO mice had significantly fewer tumors in their small intestine than WT mice (51.0±4.9 vs. 76.87±7.4 tumors per mouse, respectively; p = 0.0054, fig 4B). Differences in the number of tumors between genotypes were most pronounced in the medial (16.83±1.91 vs. 27.87±3.6 tumors per mouse; p = 0.0081) and distal (27.06±2.77 vs. 40.83±4.55 tumors per mouse; p = 0.0122) segments of the small intestine (fig 4C). Moreover, MCH-KO mice had smaller lesions than WT mice, especially in the groups of larger polyps (fig 4D). Supporting the macroscopic analysis at sacrifice, histologic analysis of small intestinal adenomas in the medial and distal segments of the small intestine confirmed the macroscopic results. Importantly, the number of high-grade adenomas was significantly lower in MCH-KO mice compared to WT mice (14.44±2.19 vs. 23.53±3.46, respectively; p = 0.0289; fig 4E and 4F).

### MCH-deficiency is Associated with Increased Apoptosis in Intestinal Adenomas

**Figure 5:**
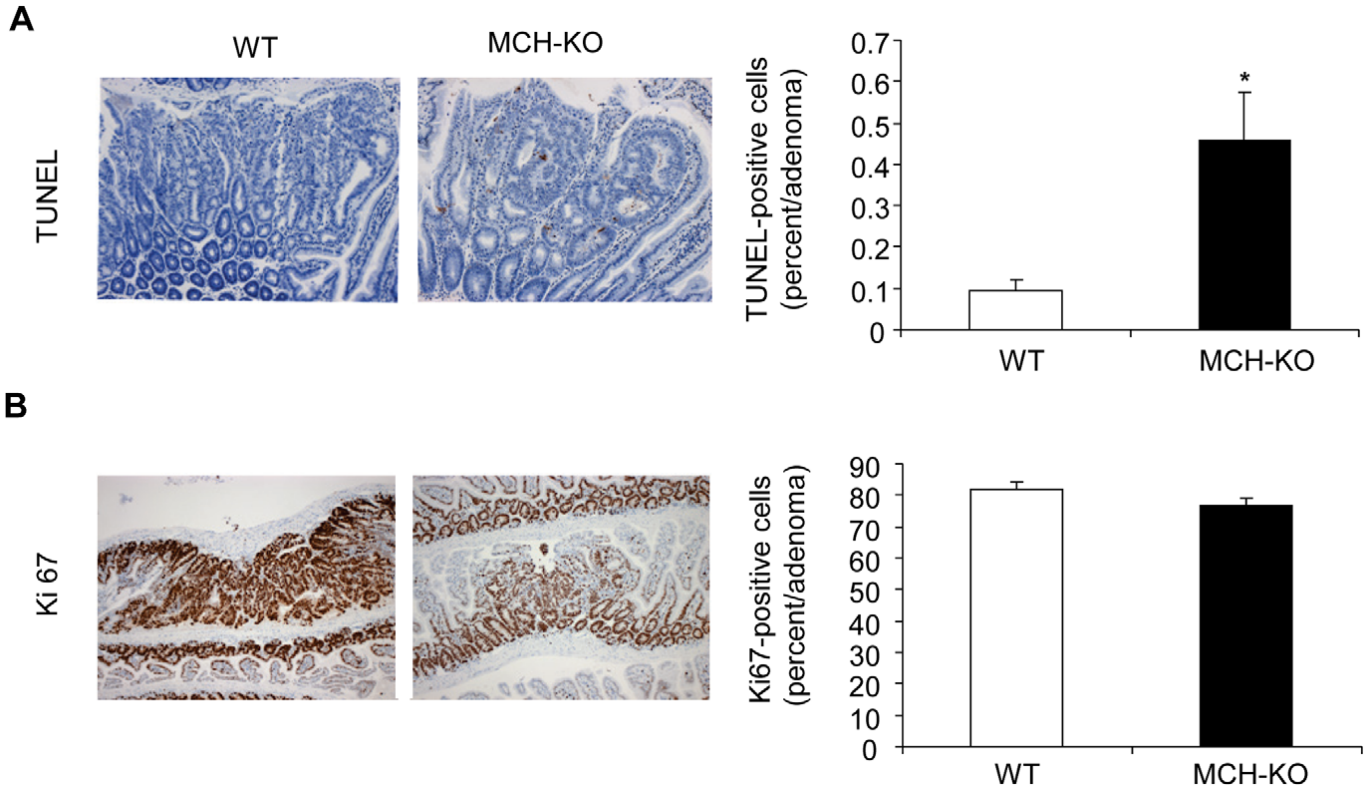
Increased apoptosis in intestinal adenomas developed in MCH-deficient APCmin mice. Paraffin sections of intestinal adenomas from WT and MCH-KO mice, both of the APCmin background, were stained for (A) TUNEL, a marker of apoptosis; and (B) Ki67, a marker of proliferation. Quantification of differences between the groups (percentage of positive cells per adenoma) is shown in graphs on the right of each picture. *p<0.05.

There are several potential mechanisms by which MCH could influence tumor development in the APCmin mice. The fact that MCH-KO mice developed fewer and smaller adenomas prompted us to examine the effects of MCH on pathways regulating cell growth and survival. Indeed, TUNEL analysis revealed that intestinal adenomas developed in MCH deficient mice had 5-fold as many apoptotic cells compared to WT mice (0.092±0.28 vs 0.458±0.117; MCH-KO vs WT, respectively; p = 0.021; fig 5A). These results are consistent with our *in vitro* findings of MCH increasing cell apoptosis in Caco2 colonic adenocarcinoma cells (fig 3C).

Furthermore, there was a trend of reduced cell proliferation in the adenomas developed in the MCH-KO mice, as revealed by Ki67 staining (77.2±2.2 vs 82.1±1.9; MCH-KO vs WT, respectively; p = 0.12; fig 5B), again consistent with our in vitro studies (fig 3D).

### Attenuated Activation of the wnt/beta-catenin and erk1/2 Signaling Pathways in APCmin Mice Lacking MCH

**Figure 6:**
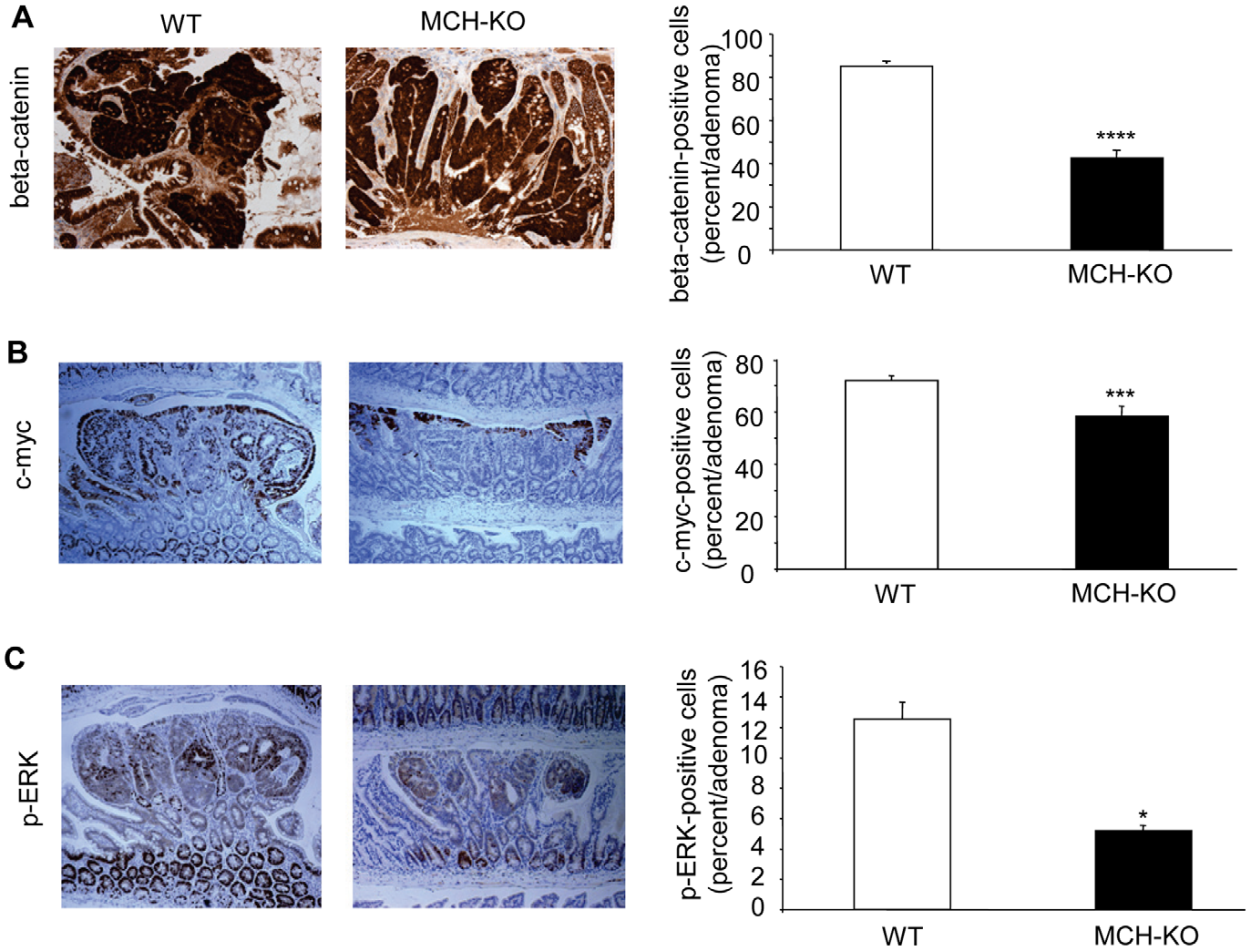
Attenuated activation of the wnt/beta-catenin and ERK signaling pathways in APCmin mice lacking MCH. Paraffin sections of intestinal adenomas from WT and MCH KO mice, both of the APCmin background, were stained for (A) beta-catenin, a downstream effector of the wnt signaling pathway; (B) c-myc, a target of beta-catenin; and (C) p-erk. Quantification of differences between the groups (percentage of positive cells per adenoma) is shown in graphs on the right of each picture. Results are expressed as mean±sem. ***p<0.005; ****p<0.001.

Nuclear accumulation of beta-catenin and expression of its downstream target c-myc [14], were determined by immunohistochemistry, and were found to be more pronounced in the WT mice compared to MCH-KO mice. Specifically, in the WT mice, on average 85.9±1.2% of cells per adenoma showed nuclear accumulation of beta-catenin, while only 42.3±3.3% in MCH-KO mice (p<0.0001, fig 6A). This difference was apparent in adenomas, but not the surrounding normal tissue. In line with this finding, 71.9±1.7% of cells in adenomas from WT mice expressed c-myc but only 58.3±3.7% in MCH-KO mice (p = 0.0011, fig 6B).

A recent report indicates that p-erk1/2 plays a critical role in stabilizing c-myc and promoting tumorigenesis through decreased apoptosis and increased proliferation [23]. Hence, we examined by immunohistochemistry erk1/2 phosphorylation in tumors from WT and MCH-KO mice. In WT mice, the majority of tumors had strong nuclear staining for p-erk within small parts of the adenoma, whereas MCH-KO mice often lacked p-erk1/2 staining or showed very few cells with nuclear staining in an adenoma (p = 0.0472, fig 6C). These results are consistent with the direct activation of erk1/2 in colonocytes in response to MCH treatment, as shown in fig. 3D.

### MCH-deficiency Attenuates DSS-induced Colorectal Tumor Development in APCmin Mice

**Figure 7:**
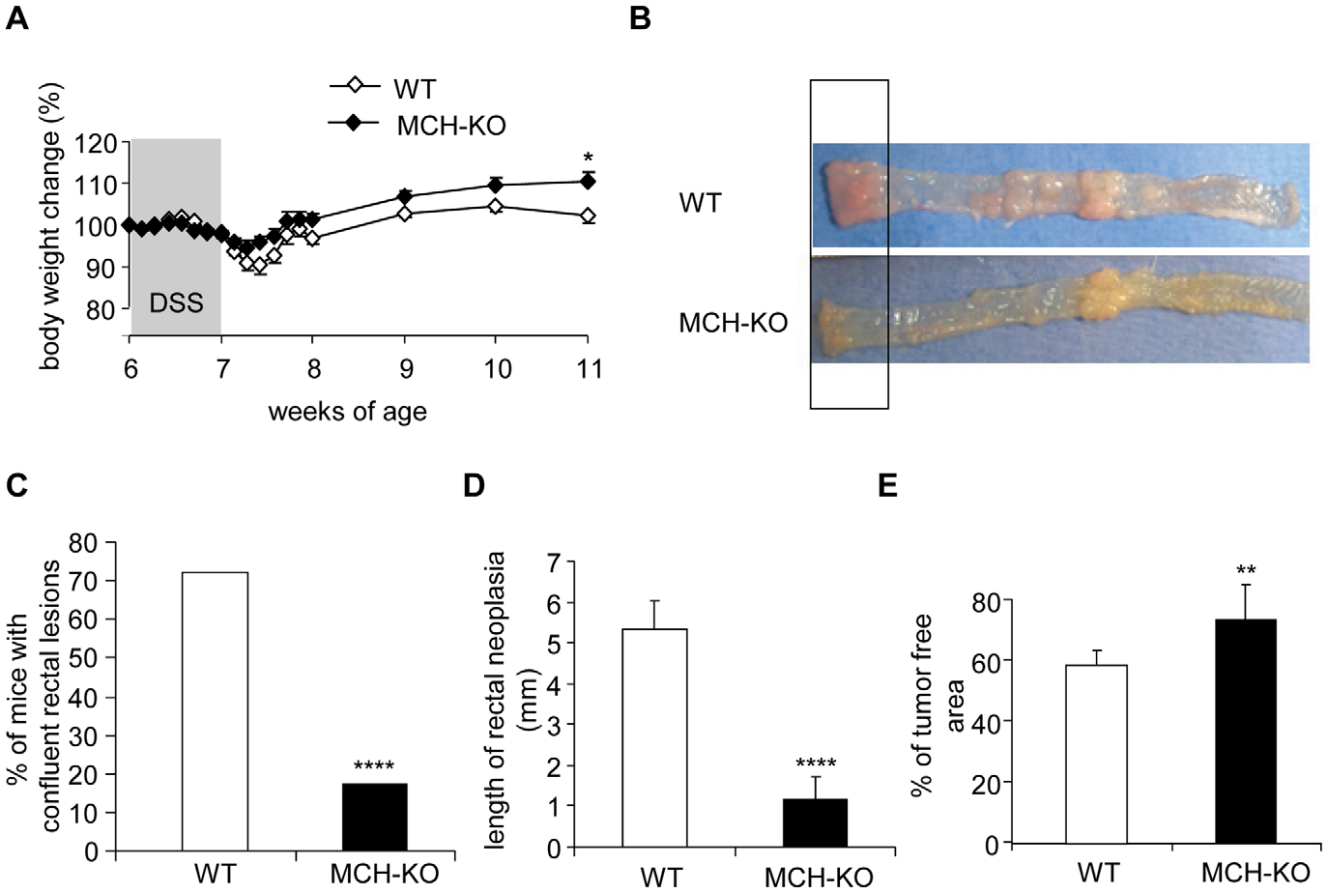
Reduced incidence and size of colorectal adenomas in DSS-treated APCmin lacking MCH. To induce colitis, six week old WT (n = 24) and MCH-KO (n = 32) mice, both of the APCmin background, were treated with 2% DSS in their drinking water for 7 days. At 11 weeks of age, mice were evaluated for colonic tumor development. (A) Changes in body weight over time in mice treated with DSS are expressed as % of their body weight at 6 weeks of age. (B) Representative pictures of tumor development patterns in the colon of WT and MCH-KO mice. Striking differences in tumor development between the two genotypes were noted in the rectal area. (C) Not only a lower percentage of MCH KO mice developed colorectal lesions, but (D) also the size of these lesions was smaller in the MCH-KO mice. (E) Percentage of adenoma-free area in the colon of WT and MCH-KO mice. *p<0.05; **p<0.01; ****p<0.001.

In the presence of dextran sodium sulphate (DSS), an irritant that causes colonic inflammation, APCmin mice develop in a short period of time multiple colonic neoplasms [19]. We induced colitis in six-week-old MCH-KO mice and their WT littermates by treating them with DSS for 7 consecutive days. Colonic tumor development was evaluated when mice were 11 weeks of age. At this point, and compared to their initial body weight, WT mice lost slightly more weight compared to MCH-KO mice (110.52±1.79 vs. 102.25±2.19% respectively, p = 0.0132; fig 7A). The majority of WT mice showed multiple individual lesions with clustering in the middle of the colon and near the rectum. In these mice, the rectal tumors were so dense and big that they became indistinguishable from one another (fig 7B). However, only a few of the MCH-KO mice developed confluent rectal tumors (23/32 vs. 4/24, respectively, p = 0.00004; fig 7C). Moreover, in those MCH-KO mice with confluent rectal tumors, the length of the tumor occupied area was shorter than in WT mice (1.15±0.58 mm vs. 5.34±0.68 mm; p<0.0001; fig 7D). Since no individual tumors could be clearly distinguished in some of the cases, the percentage of colon covered by adenomas was evaluated. MCH-deficient APCmin mice had a significant increase in tumor-free area in their colon, compared to their wild-type littermates (73.54±11.27% vs. 58.75±4.81%, respectively, p = 0.0077; fig 7E). Histologically, all adenomas showed high-grade dysplasia, but no invasive carcinoma was detected in either group. Furthermore, the adenomas from both groups showed mild infiltration of inflammatory cells, but no active colitis was detected. Thus, we found no indication of differences in the resolution of colitis between MCH-KO and WT mice at sacrifice. Additionally, at this time point, no differences in tumor development between WT and MCH-KO mice were apparent in the small intestine.

## Discussion

The role of MCH beyond the central nervous system has not yet been fully investigated. Studies from our group and others have identified the intestine as a significant source of MCH as well as a target site for its actions [3], [5], [6]. In the present study, using a compound genetic model of spontaneous intestinal tumorigenesis, we describe for the first time that mice lacking MCH in the APCmin genetic background, develop fewer and smaller adenomas in their intestine and colon. The significance of this observation is corroborated by our observation of MCH receptor positive human colonic adenocarcinomas.

One intriguing and unexpected finding in the present study is the demonstration of MCHR1 expression in LGR5-positive crypt stem cells, which are considered to play a key role in the development of intestinal tumors [20]. The implications of this observation remain to be seen. In particular for the APC model, it has been shown that deletion of APC specifically in LGR5-positive cells is sufficient to give rise to adenomas within 3–5 weeks. In contrast, deletion of APC in short lived transit amplifying cells does not promote tumorigenesis [20].

Overall, our analysis of tumor development in APCmin mice lacking MCH suggests that MCH ablation affects primarily tumor growth rather than tumor formation. Several points of evidence support this conclusion. The most pronounced difference in tumor development in the MCH-KO mice was a reduction in the number of larger adenomas, whereas the number of small tumors remained comparable between the MCH-KO and WT mice as shown in fig. 4D. Furthermore, only the number of high-grade adenomas was lower in the MCH-KO mice (fig. 4F). These observations are consistent with a direct role for MCH in promoting cell survival, as our in vitro studies also indicate (fig. 3). Indeed, this is the first report demonstrating that MCH directly inhibits cell apoptosis. The exact signaling pathways leading to this effect need to be further elucidated. However, a previous study from our group has demonstrated increased levels of the tumor suppressor p53 in the liver and spleen of MCH knockout mice [24], which is consistent with the reduced size of adenomas in the MCH deficient mice reported in the current study. Moreover, it has previously been shown that in APCmin mice, deficiency of p53 enhances the number and invasiveness of the intestinal tumors [25].

We also report here that adenomas developed in MCH-deficient mice had lower expression of c-myc, compared to wild-type mice, most likely due to reduced activation of the wnt/beta-catenin pathway. C-myc is an oncogene that plays a critical role in the early stages of carcinogenesis following inactivation of APC. Besides transcriptional upregulation of c-myc by beta-catenin, it has been shown that c-myc deficiency can rescue the phenotype of mice with APC loss, even in the presence of high levels of nuclear beta-catenin [15]. Moreover, a recent study underscores the role of erk signaling in the APCmin model of intestinal tumorigenesis by demonstrating that erk can phosphorylate and stabilize c-myc, thus preventing its ubiquitination and proteasomal degradation [23]. Indeed, it has been previously shown in different cell types that activation of MCHR1 results in erk1/2 phosphorylation [21], [22].

Previous studies have shown that MCH can act in combination with additional factors, for example forskolin or isoproterenol, to synergistically activate erk [21]. This might explain why in our *in vitro* experiments we were able to detect an MCH-mediated increase in cell proliferation only in the presence of IGF-1. The significance of this observation is underscored by epidemiologic and experimental evidence suggesting that IGF-1 signaling is critical in the pathogenesis of intestinal carcinogenesis [26]. It has been suggested that one of the mechanisms by which IGF-1 contributes to cell transformation is the phosphorylation of beta-catenin [27]. As such, agents targeting IGF-1 are currently under development for the treatment of colorectal cancer [28], and perhaps could be used in combination with anti-MCH treatments.

From a mechanistic point of view, this report has focused on the role of MCH on colonic epithelial cells. However, additional cell types might mediate the effects of MCH in tumorigenesis. For instance, the presence of MCHR1 on monocytes and T-cells has been previously recognized, though the biological significance of such findings remains elusive [29]. Interestingly, in activated lymphocytes, MCH appears to inhibit cell proliferation [29], [30]. MCH is also produced by human microvascular endothelial cells, which might be of importance in tumor angiogenesis [31].

The concept of gut neuropeptides and hormones like MCH, modulating carcinogenesis does not appear to be unique for MCH. It has been previously shown that, like MCH, gastrin-deficient APCmin mice exhibited significant decrease in the number and proliferative capacity of intestinal adenomas [32]. On the other hand, neuropeptides known to regulate cell growth, differentiation and survival, like GLP-2, do not necessarily modify intestinal tumor growth [33].

Often, chronic inflammation predisposes to cancer development via multiple mechanisms [34] and epidemiological evidence suggests that patients with inflammatory bowel disease (IBD) are at increased risk for colorectal cancer. A recent report estimated that incidence rates of cancer in patients with IBD was 75 per 100,000 person years compared to 47 per 100,000 person-years in patients without IBD [35]. Notably, in patients with IBD we have found a significant upregulation of MCH and its receptor, which correlated positively with the severity of the disease [5]. In this context, MCH not only promotes intestinal inflammation as we have previously demonstrated [5], [6], which is a predisposing factor for tumorigenesis, but also, based on the present study, it contributes to intestinal tumor growth independently of inflammation. Combined, these properties render MCH an attractive target for the treatment of inflammatory bowel disease, which will also reduce the risk of subsequent cancer development. Indeed, during the last decade there is an ongoing investigation by many pharmaceutical companies on MCH antagonists, primarily for the treatment of obesity [1]. The fields of IBD and cancer therapeutics are likely to benefit in the foreseeable future by taking advantage of this momentum.

## References

[1] Chung S, Parks GS, Lee C, Civelli O (2011) Recent updates on the melanin-concentrating hormone (MCH) and its receptor system: lessons from MCH1R antagonists. J Mol Neurosci 43: 115–121. 20582487 10.1007/s12031-010-9411-4 PMC3018593

[2] Hervieu G, Nahon JL (1995) Pro-melanin concentrating hormone messenger ribonucleic acid and peptides expression in peripheral tissues of the rat. Neuroendocrinology 61: 348–364. 7783849 10.1159/000126857

[3] Hervieu G, Volant K, Grishina O, Descroix-Vagne M, Nahon JL (1996) Similarities in cellular expression and functions of melanin-concentrating hormone and atrial natriuretic factor in the rat digestive tract. Endocrinology 137: 561–571. 8593803 10.1210/endo.137.2.8593803

[4] Burdyga G, Varro A, Dimaline R, Thompson DG, Dockray GJ (2006) Feeding-dependent depression of melanin-concentrating hormone and melanin-concentrating hormone receptor-1 expression in vagal afferent neurones. Neuroscience 137: 1405–1415. 16359819 10.1016/j.neuroscience.2005.10.057

[5] Kokkotou E, Moss AC, Torres D, Karagiannides I, Cheifetz A, et al (2008) Melanin-concentrating hormone as a mediator of intestinal inflammation. Proc Natl Acad Sci U S A 105: 10613–10618. 18650383 10.1073/pnas.0804536105 PMC2492477

[6] Kokkotou E, Espinoza DO, Torres D, Karagiannides I, Kosteletos S, et al (2009) Melanin-concentrating hormone (MCH) modulates C difficile toxin A-mediated enteritis in mice. Gut 58: 34–40. 18824554 10.1136/gut.2008.155341 PMC3058236

[7] Zhao D, Zhan Y, Zeng H, Koon HW, Moyer MP, et al (2005) Neurotensin stimulates interleukin-8 expression through modulation of I kappa B alpha phosphorylation and p65 transcriptional activity: involvement of protein kinase C alpha. Mol Pharmacol 67: 2025–2031. 15755906 10.1124/mol.104.010801

[8] Koon HW, Zhao D, Na X, Moyer MP, Pothoulakis C (2004) Metalloproteinases and transforming growth factor-alpha mediate substance P-induced mitogen-activated protein kinase activation and proliferation in human colonocytes. J Biol Chem 279: 45519–45527. 15319441 10.1074/jbc.M408523200

[9] Coussens LM, Werb Z (2002) Inflammation and cancer. Nature 420: 860–867. 12490959 10.1038/nature01322 PMC2803035

[10] White BD, Chien AJ, Dawson DW (2012) Dysregulation of Wnt/beta-Catenin Signaling in Gastrointestinal Cancers. Gastroenterology 142: 219–232. 22155636 10.1053/j.gastro.2011.12.001 PMC3285553

[11] Taketo MM, Edelmann W (2009) Mouse models of colon cancer. Gastroenterology 136: 780–798. 19263594 10.1053/j.gastro.2008.12.049

[12] Powell SM, Zilz N, Beazer-Barclay Y, Bryan TM, Hamilton SR, et al (1992) APC mutations occur early during colorectal tumorigenesis. Nature 359: 235–237. 1528264 10.1038/359235a0

[13] Korinek V, Barker N, Morin PJ, van Wichen D, de Weger R, et al (1997) Constitutive transcriptional activation by a beta-catenin-Tcf complex in APC−/− colon carcinoma. Science 275: 1784–1787. 9065401 10.1126/science.275.5307.1784

[14] He TC, Sparks AB, Rago C, Hermeking H, Zawel L, et al (1998) Identification of c-MYC as a target of the APC pathway. Science 281: 1509–1512. 9727977 10.1126/science.281.5382.1509

[15] Sansom OJ, Meniel VS, Muncan V, Phesse TJ, Wilkins JA, et al (2007) Myc deletion rescues Apc deficiency in the small intestine. Nature 446: 676–679. 17377531 10.1038/nature05674

[16] Su LK, Kinzler KW, Vogelstein B, Preisinger AC, Moser AR, et al (1992) Multiple intestinal neoplasia caused by a mutation in the murine homolog of the APC gene. Science 256: 668–670. 1350108 10.1126/science.1350108

[17] Dietrich WF, Lander ES, Smith JS, Moser AR, Gould KA, et al (1993) Genetic identification of Mom-1, a major modifier locus affecting Min-induced intestinal neoplasia in the mouse. Cell 75: 631–639. 8242739 10.1016/0092-8674(93)90484-8

[18] Wasan HS, Novelli M, Bee J, Bodmer WF (1997) Dietary fat influences on polyp phenotype in multiple intestinal neoplasia mice. Proc Natl Acad Sci U S A 94: 3308–3313. 9096389 10.1073/pnas.94.7.3308 PMC20365

[19] Tanaka T, Kohno H, Suzuki R, Hata K, Sugie S, et al (2006) Dextran sodium sulfate strongly promotes colorectal carcinogenesis in Apc(Min/+) mice: inflammatory stimuli by dextran sodium sulfate results in development of multiple colonic neoplasms. Int J Cancer 118: 25–34. 16049979 10.1002/ijc.21282

[20] Barker N, Ridgway RA, van Es JH, van de Wetering M, Begthel H, et al (2009) Crypt stem cells as the cells-of-origin of intestinal cancer. Nature 457: 608–611. 19092804 10.1038/nature07602

[21] Pissios P, Trombly DJ, Tzameli I, Maratos-Flier E (2003) Melanin-concentrating hormone receptor 1 activates extracellular signal-regulated kinase and synergizes with G(s)-coupled pathways. Endocrinology 144: 3514–3523. 12865333 10.1210/en.2002-0004

[22] Bradley RL, Mansfield JP, Maratos-Flier E, Cheatham B (2002) Melanin-concentrating hormone activates signaling pathways in 3T3-L1 adipocytes. Am J Physiol Endocrinol Metab 283: E584–592. 12169453 10.1152/ajpendo.00161.2002

[23] Lee SH, Hu LL, Gonzalez-Navajas J, Seo GS, Shen C, et al (2010) ERK activation drives intestinal tumorigenesis in Apc(min/+) mice. Nat Med 16: 665–670. 20473309 10.1038/nm.2143 PMC2882530

[24] Jeon JY, Bradley RL, Kokkotou EG, Marino FE, Wang X, et al (2006) MCH−/− mice are resistant to aging-associated increases in body weight and insulin resistance. Diabetes 55: 428–434. 16443777 10.2337/diabetes.55.02.06.db05-0203

[25] Halberg RB, Katzung DS, Hoff PD, Moser AR, Cole CE, et al (2000) Tumorigenesis in the multiple intestinal neoplasia mouse: redundancy of negative regulators and specificity of modifiers. Proc Natl Acad Sci U S A 97: 3461–3466. 10716720 10.1073/pnas.050585597 PMC16262

[26] Guo YS, Narayan S, Yallampalli C, Singh P (1992) Characterization of insulinlike growth factor I receptors in human colon cancer. Gastroenterology 102: 1101–1108. 1312970

[27] Playford MP, Bicknell D, Bodmer WF, Macaulay VM (2000) Insulin-like growth factor 1 regulates the location, stability, and transcriptional activity of beta-catenin. Proc Natl Acad Sci U S A 97: 12103–12108. 11035789 10.1073/pnas.210394297 PMC17301

[28] Adachi Y, Yamamoto H, Ohashi H, Endo T, Carbone DP, et al (2010) A candidate targeting molecule of insulin-like growth factor-I receptor for gastrointestinal cancers. World J Gastroenterol 16: 5779–5789. 21154998 10.3748/wjg.v16.i46.5779 PMC3001968

[29] Lakaye B, Coumans B, Harray S, Grisar T (2009) Melanin-concentrating hormone and immune function. Peptides 30: 2076–2080. 19450627 10.1016/j.peptides.2009.05.004

[30] Verlaet M, Adamantidis A, Coumans B, Chanas G, Zorzi W, et al (2002) Human immune cells express ppMCH mRNA and functional MCHR1 receptor. FEBS Lett 527: 205–210. 12220661 10.1016/s0014-5793(02)03232-5

[31] Orihara K, Morita H, Yagami A, Kajiwara N, Nakae S, et al (2009) TH2 cytokines potently induce an appetite-stimulating peptide, melanin-concentrating hormone, in human vascular endothelial cells. J Allergy Clin Immunol 124: 612–614, 614 e611–612. 19541358 10.1016/j.jaci.2009.04.039

[32] Koh TJ, Bulitta CJ, Fleming JV, Dockray GJ, Varro A, et al (2000) Gastrin is a target of the beta-catenin/TCF-4 growth-signaling pathway in a model of intestinal polyposis. J Clin Invest 106: 533–539. 10953028 10.1172/JCI9476 PMC380254

[33] Koehler JA, Harper W, Barnard M, Yusta B, Drucker DJ (2008) Glucagon-like peptide-2 does not modify the growth or survival of murine or human intestinal tumor cells. Cancer Res 68: 7897–7904. 18829546 10.1158/0008-5472.CAN-08-0029 PMC3606135

[34] O’Connor PM, Lapointe TK, Beck PL, Buret AG (2010) Mechanisms by which inflammation may increase intestinal cancer risk in inflammatory bowel disease. Inflamm Bowel Dis 16: 1411–1420. 20155848 10.1002/ibd.21217

[35] Herrinton LJ, Liu L, Levin TR, Allison JE, Lewis JD, et al (2012) Incidence and Mortality of Colorectal Adenocarcinoma in Persons with Inflammatory Bowel Disease from 1998 to 2010. Gastroenterology. 10.1053/j.gastro.2012.04.054 22609382

